# SUNSPACE, A Porous Material to Reduce Air Particulate Matter (PM)

**DOI:** 10.3389/fchem.2018.00534

**Published:** 2018-10-30

**Authors:** Alessandra Zanoletti, Fabjola Bilo, Laura Borgese, Laura E. Depero, Ario Fahimi, Jessica Ponti, Andrea Valsesia, Rita La Spina, Tiziano Montini, Elza Bontempi

**Affiliations:** ^1^INSTM and Department of Mechanical and Industrial Engineering, University of Brescia, Brescia, Italy; ^2^European Commission, Directorate General Joint Research Centre, Directorate F—Health, Consumers and Reference Materials, Consumer Products Safety Unit (F.2), Ispra, Italy; ^3^Department of Chemical and Pharmaceutical Sciences, CNR-ICCOM URT and INSTM Trieste Research Unit, University of Trieste, Trieste, Italy

**Keywords:** SUNSPACE, azure chemistry, porous material, leaf, air particulate matter (PM) capture, nanoparticles

## Abstract

The World Health Organization reports that every year several million people die prematurely due to air pollution. Poor air quality is a by-product of unsustainable policies in transportation, energy, industry, and waste management in the world's most crowded cities. Particulate matter (PM) is one of the major element of polluted air. PM can be composed by organic and inorganic species. In particular, heavy metals present in PM include, lead (Pb), mercury (Hg), cadmium, (Cd), zinc (Zn), nickel (Ni), arsenic (As), and molybdenum (Mo). Currently, vegetation is the only existing sustainable method to reduce anthropogenic PM concentrations in urban environments. In particular, the PM-retention ability of vegetation depends on the surface properties, related to the plant species, leaf and branch density, and leaf micromorphology. In this work, a new hybrid material called SUNSPACE (SUstaiNable materials Synthesized from by-Products and Alginates for Clean air and better Environment) is proposed for air PM entrapment. Candle burning tests are performed to compare SUNSPACE with *Hedera Helix L*. leafs with respect to their efficacy of reducing coarse and fine PM. The temporal variation of PM_10_ and PM_2.5_ in presence of the trapping materials, shows that *Hedera Helix L*. surface saturates more rapidly. In addition, the capability of SUNSPACE in ultrafine PM trapping is also demonstrated by using titanium dioxide nanoparticles with 25 nm diameter. Scanning electron microscope (SEM) and Transmission electron microscope (TEM) images of SUNSPACE after entrapment tests highlight the presence of collected nanoparticles until to about 0.04 mm in depth from the sample surface. N_2_ physisorption measurements allow to demonstrate the possibility to SUNSPACE regeneration by washing.

## Introduction

Particulate matter (PM) has been contributing to air pollution for decades if not centuries. Atmospheric pollutants, due to both natural and anthropogenic sources, are seldom confined to urban and industrial centers where they are predominately produced but they are often brought thousands of kilometers away, also impacting regions far from their source. Moreover, it is fundamental to highlight that not only primary aerosols, that are PM directly emitted into the atmosphere, but also secondary aerosols exist. Secondary aerosols, as for example sulfate and nitrate, are formed in the atmosphere by chemical reactions on the primary aerosols (Crippa et al., 2013).

PM can be made of organic and inorganic species. In particular, heavy metals present in PM include, but are not limited to lead (Pb), mercury (Hg), cadmium, (Cd), zinc (Zn), nickel (Ni), arsenic (As), and molybdenum (Mo). Heavy metals are typically released into the atmosphere from industrial processes. They have been accumulating since humans first started smelting metals in significant quantities during the Bronze Age c. 5,000 years ago (Zereini and Wiseman, 2010).

Other processes that contribute to the diffusion of toxic metals into the atmosphere include the combustion of fossil fuels, the manufacturing and application of fertilizers, cement production, and wood combustion (Nriagu and Pacyna, 1988).

PM_2.5_ (aerodynamic diameter ≤2.5 μm) and/or PM_10_ (aerodynamic diameter ≤10 μm) levels exceed current standards and/or recommended values in many countries and they are alarmingly high in several big cities, particularly in some developing countries. The solution to PM increase represents a tremendous scientific challenge as well as an economic one. Indeed, the reduction of vehicular or industrial PM emissions is not always an economical viable solution in highly urbanized areas.

Therefore, political authorities will have to improve strategies to mitigate PM concentrations in cities: there is a critical need for air quality policy and emissions regulations that minimize PM exposure and health risks as much as possible.

Porous materials with large pore volume can provide spatial confinement to trap small size PM that represent the most problematic issue of air dispersed particles. Literature reports a variety of porous materials, such as metal-organic matrices, porous carbon materials, silica, and many others usable for nanoparticle inclusion (Zhu and Xu, 2016).

Silica, which has excellent thermal stability, is one of the most abundant crustal material. Silica powders, in terms of micro-silica, provide a promising support for the immobilization of particles with low dimensions (<10 μm) (Wan and Zhao, 2007). In addition, several sources of waste silica (as rice husk ash (Bosio et al., 2014) and silica fume (Benassi et al., 2017; Rodella et al., 2017; Pasquali et al., 2018) may be used to produce sustainable porous materials (Bontempi, 2017a,b).

In this context, a new porous material, called SUNSPACE (SUstaiNable materials Synthesized from by-Products and Alginates for Clean air and better Environment) (Zanoletti et al., 2018a) (Zanoletti et al., 2018b), has been recently realized and proposed as the first viable technical solution for the reduction of aero-dispersed PM. The idea is to propose a low-cost material, that can be integrable with the already existing buildings and infrastructures, with low impact.

The first characterization (Zanoletti et al., 2018b) showed that the new porous material can also adsorb pollutants present in water, then it could be also used to degrade some contaminants. In addition, if the new material is covered by a thin layer of titanium dioxide, it can also act as a catalyst for photo-degradation.

**Figure 1:**
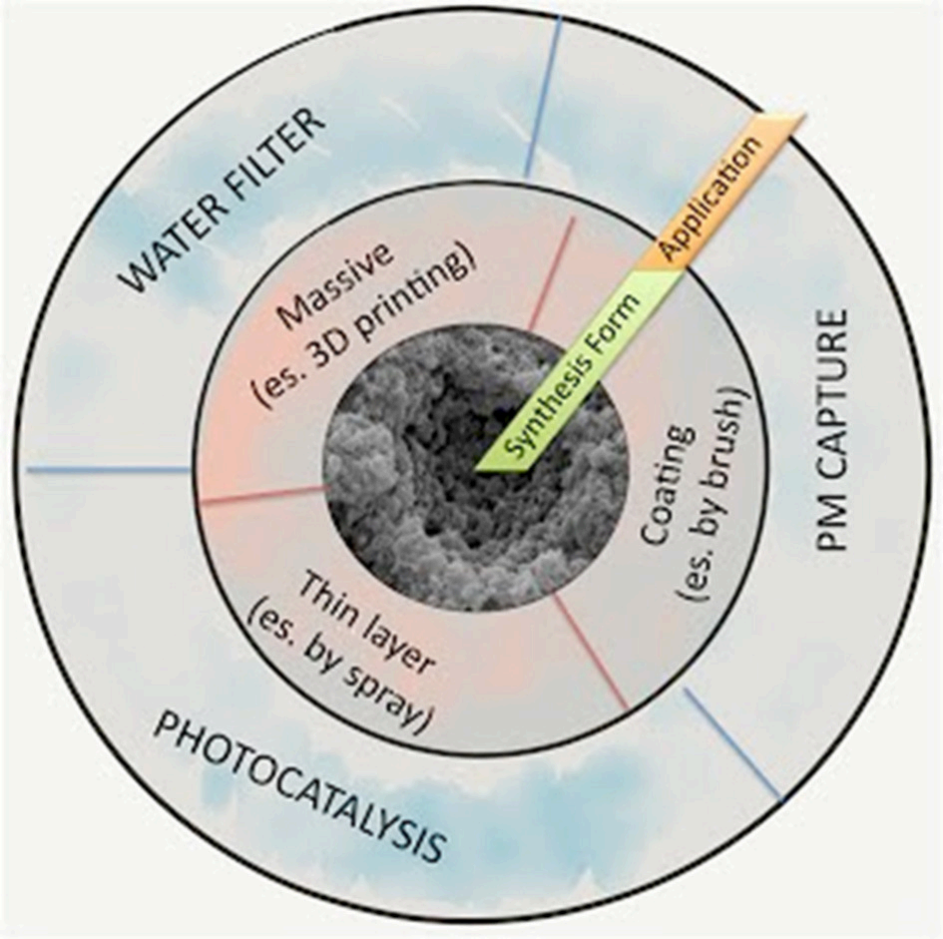
Possible SUNSPACE applications, based on the selected synthesis form (massive, coating, thin layer).

Figure 1 summarizes the possible SUNSPACE applications, based on the selected synthesis form (massive, coating, thin layer). As shown in Figure 1, SUNSPACE can be used as a coating (as a plaster on a wall or roof tiles) to reduce the PM in urban areas. Indeed, to optimize air trapping efficiency, it is fundamental to promote SUNSPACE diffusion on all the available building surfaces.

This new technology and material lay the foundation for a new vision of chemistry, proposing environmental remediation, with great attention to the sustainability of the employed synthesized material, recently proposed as “Azure Chemistry” (Zanoletti et al., 2018a).

Actually, tree planting represents the most efficient and sustainable method to reduce PM in urban areas. Indeed, dust containing heavy metals are deposited on surfaces, including plant foliage. Several studies report on foliar uptake of heavy metal from atmospheric PM deposition (Gajbhiye et al., 2016).

Leaves can act as PM traps, thus improving air quality due to their texture and large contact area (Freer-Smith et al., 1997).

Some papers have quantified the amount of PM removal from urban air by vegetation. For example, it was shown that on a larger scale, trees can annually remove approximately 300 tons of air pollutants (Cavanagh, 2008). Only in the USA, urban trees and shrubs remove about 215,000 tons PM_10_ per year (Nowak et al., 2006). In Beijing, China, trees in the city center removed 772 tons of PM_10_ in 1 year (Yang et al., 2004). Thus, tree planting can be considered a viable and sustainable pollution mitigation measure in a variety of urban settings (Chen et al., 2017).

However, there are several limitations and barriers to achieve air PM trapping by urban greening only, including (but not limited to): prevailing soil conditions, space utilization, architectural design, sub-surface infrastructure, availability of sunlight and the size of some trees compared to the adjacent buildings (Johnston and Newton, 2004).

In addition, leaves cannot survive in winter. Finally, the effectiveness of vegetation as a long-term alternative to other PM reduction media is still under debate. Indeed, most of the particles are retained on the plant surface and subsequently removed from the canopies by resuspension to the atmosphere (Chen et al., 2017).

For these reasons, the leaves activity should be improved by the use of additional low-cost media, and in this context a complementary viable solution can be SUNSPACE.

In this paper, the ability of the new porous material in fine PM trapping is presented in comparison to a *Hedera Helix L*. leaf one of the most studied leaf on this purpose. In particular, *Hedera Helix L*. has shown relatively high PM densities accumulation in all size fractions due to the important role of the surface wax (Rodrigues et al., 2015; Weerakkody et al., 2017).

Several papers in literature report the foliage capability of PM trapping (Weerakkody et al., 2017), but at the best of our knowledge, no comparison with filters or other material designed on this purpose can be found.

In this paper the SUNSPACE capability in coarse (PM_2.5−10_) and fine (PM_2.5_) PM entrapment is evaluated by means of candle burning tests, with respect to *Hedera Helix L*. Candle burning was chosen as a pollution source, due to the prevalence of fine (and ultrafine) PM emission and some studies have shown that candles can be sources of heavy metals, such as Pb, Zn, and Ni (Pedersen et al., 2017).

In addition, the SUNSPACE capability in ultrafine PM (PM with diameter <0.1 μm) entrapment is here demonstrated by electron microscopy analysis after exposure to titanium dioxide nanoparticles considered as a positive control for our system material. In this case, they were used to investigate the particles penetration depth in the porous material.

Titanium dioxide (JRCNM1005a) was selected due to the industrial interest in producing it for many different advanced nano-technological applications (Environment Directorate, 2016).

The aim of this work is to show that SUNSPACE, specifically designed for PM entrapment, is comparable to leaves in fine PM capture efficacy, and ultrafine PM entrapment is also possible. As a consequence, SUNSPACE is proposed as a synergic component, to be coupled for example with vegetation, to improve the air quality in urban environments.

## Experimental section

### Materials and characterization

Calcium iodate (Ca(IO_3_)_2_, CAS number: 7789-80-2), sodium alginate (SA, CAS number: 9005-38-3), sodium bicarbonate (NaHCO_3_, CAS number 14455-8, ≥99.8% w/w) were purchased from Sigma Aldrich. Titanium dioxide (JRCNM1005a—old OECD nomenclature: NM-105) was supplied by the JRC Nanomaterials Repositor (Rasmussen et al., 2014; Environment Directorate, 2016).

For samples preparation, food grade sodium bicarbonate was bought in a local store. Silica fume was provided by Metalleghe SPA, Brescia, Italy, as an industrial by-product derived from ferrosilicon and silicon metal alloy processing.

MilliQ water (Millipore DirectQ-5 purification system) was used for the preparation of various solutions.

For TEM analysis 1 mg/mL suspension of silica fume was prepared in MilliQ water and 3 μL of 1/2 dilution was deposited on Formvar Carbon coated 200 mesh copper grids (Agar Scientific, USA) and dehydrated overnight in desiccator before TEM (JEOL-JEM 2100; JEOL, Italy) analysis at 120 kV.

SUNSPACE material was sonicated for 10 min in 1 mL MilliQ water and 3 μL deposited on grid and analyzed or embedded in epoxy resin solution (Sigma Aldrich, Italy) and sample cut in ultrathin sections (50–70 nm) by Leica UCT ultramicrotome (Leica, Italy).

The elements identification was done in TEM mode at 120 kV by Energy Dispersive X-ray analysis (EDX) using X-Flash Detector 5030 (Brüker, Italy) coupled with the microscope.

Quantitative analysis was automatically obtained by Cliff-Lorimer model (Cu-C-deconvoluted TEM mode) using QUANTAX 200 software (Brüker, Italy). Nitrogen (N_2_) physisorption (Micromeritics ASAP 2020 analyzer) measurements at the liquid nitrogen temperature were used to investigate the textural properties of the materials. Prior measurements 500 mg of each sample were degassed at 100°C overnight.

### Porous material synthesis

The raw materials and the synthesis technique for SUNSPACE were settled on the basis of multi-criteria decision analysis, recently published (Zanoletti et al., 2018a).

Porous material was synthesized by mixing vigorously 25 mL of milliQ water with 0.6 g of sodium alginate, as a gelling agent. Afterwards 1 g of calcium iodate was added to the solution and energetically mixed until the gel is formed. Then 17.88 g of silica fume (corresponding to 72% w/w of solid content) was added and finally 5 g of sodium bicarbonate. The slurry obtained was inserted in a mold and put on a heating plate for 1 h at 70/80°C. At this temperature sodium bicarbonate decomposed and released CO_2_ giving the space for pore formation. Then the material was left at room temperature for 1 week for consolidation. After synthesis the samples were rinsed with milliQ water to remove iodine salts, that could occlude the SUNSPACE pores (Zanoletti et al., 2018b). This is defined the pristine porous material.

For comparison in PM trapping a *Hedera Helix L*. leaf was selected and rinsed with milliQ water before the experiments.

### Candle burning tests

The fine PM trapping tests were realized in a box (made in polyethylene terephthalate, with a volume of about 10 L) designed to contain the emission source (a candle), a particles counting (AirVisual for PM_10_ and PM_2.5_), and a material that could accumulate PM (SUNSPACE or a *Hedera Helix L*. leaf).

All the experiments were performed on the same environmental conditions (temperature of about 23°C, pressure of about 1 atm), to compare the obtained results. The experimental set-up, used in this work, was the outcome of several tests. Different conditions were tested, before to obtain the best experiment setup.

The same PM source was used to evaluate the PM removal of a leaf and SUNSPACE: all tests were made by using the same candle typology to obtain the PM emission repeatability. In each test, a candle was lighted for 10 s, extinguished by covering it with an aluminum sheet for 5 s, before to insert it in the box. Over the PM source a trapping material (SUNSPACE and *Hedera Helix L*. leaf) was placed at about 10 cm from the emitting source. The trapping material surface was about 35 cm^2^. The particle counting quantified the PM_10_ (coarse PM) and PM_2.5_ (fine PM) concentration in the box, due to the emitting source. The PM data were recorded for 15 min.

The first test was made with only the emitting source (without any trapping material). The tests with the two trapping materials were repeated twice (Test 1 and Test 2) to verify the capability of the materials to accumulate PM.

### Titanium dioxide trapping test and electron microscopy analysis

A cylindrical pristine SUNSPACE sample (thickness of 0.5 cm and a diameter of 2 cm and weight 1.2 g) was exposed to 10 mg of titanium dioxide powder directly deposited on SUNSPACE porous material and manually shaken for 10 s in a closed plastic bag.

After exposure, a piece of SUNSPACE sample was cut in the central part and washed in 1 mL MilliQ water for 5 min. The rinsed sample was sonicated for 10 min in 1 mL MilliQ water and 3 μL deposited on Formvar Carbon coated 200 mesh copper grids (Agar Scientific, USA) and dehydrated overnight in a desiccator before TEM (JEOL-JEM 2100; JEOL, Italy) analysis at 120 kV.

This sample preparation was selected instead of the embedding in epoxy resin to increase the probability to find titanium dioxide nanoparticles.

The elements identification was done in STEM mode mapping the sample at 120 kV by Energy Dispersive X-ray analysis (EDX) using X-Flash Detector 5030 (Brüker, Italy) coupled with the microscope.

SEM measurements were performed by a FEI NOVA 600 (FEI, currently Thermofisher, Eindhoven, The Netherlands), Dual Beam, using an acceleration voltage ranging from 5 to 25 KeV and acquiring secondary electrons. The images of the surface were performed at different tilting angles (0 and 45°). Images and elemental analysis of the cross section of the sample were performed at 0° tilting angle.

Energy-Dispersive X-ray Spectroscopy (EDS) were carried out *in-situ* using an EDAX analyzer (AMETEK BV, The Netherlands) with element spectral resolution and sensitivity down to the carbon element. Elemental analysis maps were obtained by acquiring first a secondary electron map at 10 KeV and the X-ray elemental spectrum of the scanned region. Then the elemental map was generated fixing the energy at the different detected elements and scanning the intensity of the generated X-rays over the field-of-view.

After TiO_2_ exposition, a piece of SUNSPACE was washed in MilliQ water, to remove the trapped nanoparticles, and to make N_2_ physisorption measurements.

## Discussion

Silica fume is an industrial by-product, originated by the production of metallic silicon or ferrosilicon in the alloys industry (Sanjuán et al., 2015).

As recently shown (Zanoletti et al., 2018a,b), the porous material called SUNSPACE is obtained by modified silica fume, that can adsorb different anions (Lei et al., 2016). The combination of negatively charged alginate groups with the positively charged silica surface, lead to a stable material. In particular, it was supposed that modified SiO_2_ surface, due to the Ca^2+^ ions addition (deriving from calcium iodate or calcium chloride), could interact with the –[COO^−^] groups of sodium alginate to promote the formation of new bonds (Zanoletti et al., 2018b). In the present paper, calcium iodate was selected because it forms a soluble salt with sodium, and it can be easily highlighted by chemical analysis.

**Figure 2:**
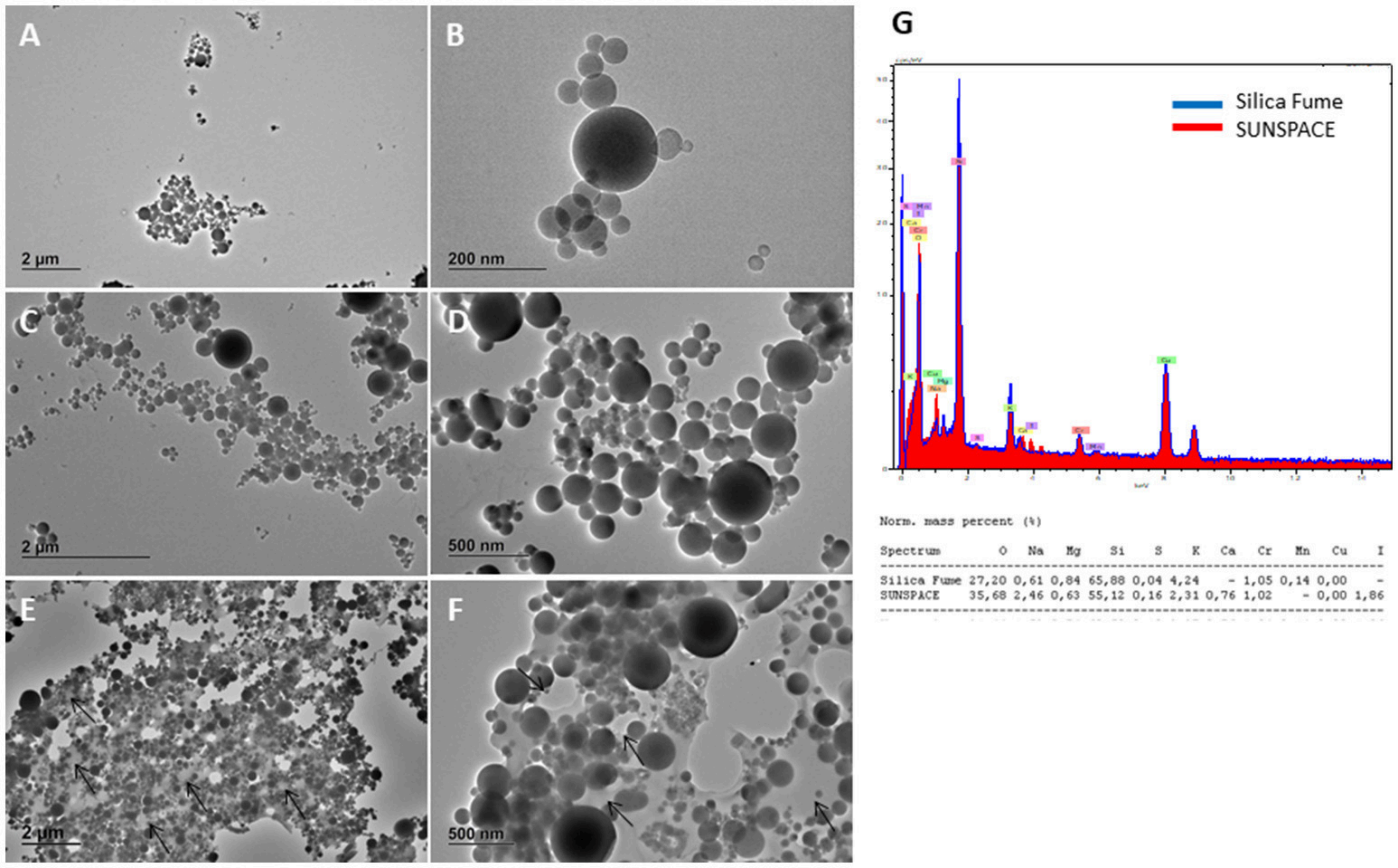
TEM images (120 kV) of silica fume sample **(A,B)** SUNSPACE prepared by sonication **(C,D)** SUNSPACE ultrathin slices **(E,F)**, and EDX spectra of Silica Fume and SUNSPACE **(G)**. Silica fume shows particles of diameter of 20–500 nm. Once embedded in epoxy resin and cut in ultrathin slices, SUNSPACE sample shows pores of 200–600 nm diameter (arrows). Elemental analysis of SUNSPACE shows presence of Iodium and Calcium not measured in silica fume sample.

TEM images of the starting silica and the obtained pristine SUNSPACE material are reported in Figure 2. Silicon dioxide powder consists of spherical-like particles with diameters generally ranging from 20 to 500 nm, according to the literature (Zhou et al., 2016). Some larger particles (with a diameter of about 2 μm) may be also found occasionally. Elemental analysis shows presence of Ca and I in SUNSPACE material and not in silica fume, as expected. Moreover, the decreased amount of Si and the increased amount of O in SUNSPACE, compare with the silica fume, is due to the functional groups derived from alginate, contributing to the formation of the new material (see Figure 2G).

TEM images reported in Figure 2 also show the silica fume morphology highlighting the difference in silica particles aggregation, before and after the synthesis of SUNSPACE. It is evident that, although the porous sample was sonicated to obtain a suitable material for TEM analysis, SUNSPACE particles aggregation agrees with the proposed structural model (Zanoletti et al., 2018b), also confirmed by elemental analysis. It also appears that the material synthesis has preserved the spherical morphology of starting silica particles, that aggregate with a good packing, where finest particles fill the voids between the largest ones. Ultrathin slice of SUNSPACE clearly shows pores of 200–600 nm diameter generated by sodium bicarbonate decomposition.

**Figure 3:**
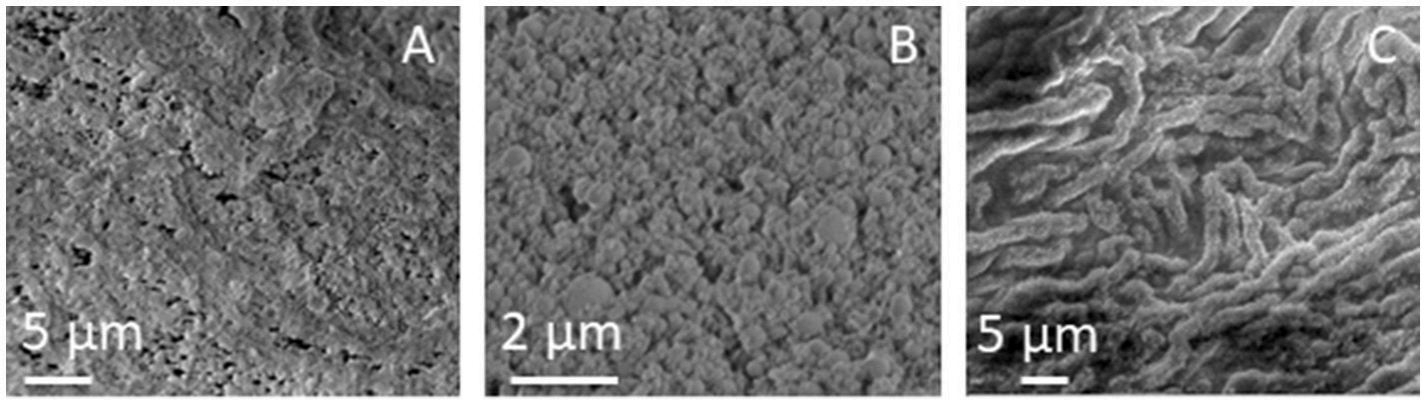
**(A,B)** SEM images of SUNSPACE, reported at different magnifications and 3 **(C)** SEM image of the *Hedera Helix L*. leaf surface.

SEM images of pristine SUNSPACE, at different magnifications, are reported in Figure 3, where the porosity is highlighted, with pores with dimensions of some μm. These pore sizes are ideal for fine PM entrapment. In a very recent paper (Zanoletti et al., 2018b) it was also reported that SUNSPACE not only contains macropores, but also mesopores. In particular, ink-bottle shaped pores are present with dimensions in the range of nanometers, as suggested by the hysteresis loops observed in the N_2_ physisorption isotherms (Zanoletti et al., 2018b).

However, it is not simple to visualize these pores, that are hypothesized to play a fundamental role in capturing fine and ultrafine PM.

The high magnification SEM image reported in Figure 3B, highlights the high aggregation of material particles, in accord with TEM results. In this image it is possible to see some dark areas, that may be attributed to pores with dimensions lower than 1 μm.

Air PM can be trapped by SUNSPACE when it passes close to its surface. It is evident that a rough and porous surface increases the probability of deposition compared with the smooth, manufactured surfaces generally present in urban areas. For example, 10–30 times faster deposition has been found for ultrafine particles on synthetic grass compared with glass and cement surfaces (Roupsard et al., 2013).

The process of removing particles or gases from the atmosphere through the delivery of mass to the surface by non-precipitation is defined as “dry deposition.” Dry deposition of air PM on vegetation, extensively studied for leaves, takes place via sedimentation under gravity, impaction (via turbulent transfer), interception, and diffusion (Wang et al., 2006).

PM removal by foliage is mainly driven by the interactions between particles and the leaf surface depending on the morphology such as shape, size, and orientation (Weerakkody et al., 2017).

Epicuticular wax is present on the leaf surface with different morphologies, such as tubules, platelets, rodlets, threads, and thin films or thicker crusts (Barthlott et al., 1998). The SEM image of the *Hedera Helix L*. surface structure, used in this study, is reported in Figure 3C. It is observed that *Hedera Helix L*. epicuticular wax is made of long tubules, with a diameter generally less than 2 μm. This is consistent with literature results, reporting the prominent structures of *Hedera Helix L*. epicuticular wax with respect to other leaves (Weerakkody et al., 2017). The voids between these structures have the right dimension for fine PM trapping.

To verify coarse and fine PM trapping capability of SUNSPACE, in comparison to a *Hedera Helix L*. leaf, some tests were performed in static conditions, as described in the experimental section. Indeed, wind speed, wind turbulence, humidity, and rainfall, all have a considerable influence on PM deposition on vegetation (Litschike and Kuttler, 2008). Static conditions are obtained in a box, where it was possible to monitor the PM concentration by a particles counter, neglecting all the contributions of atmospheric conditions.

**Figure 4:**
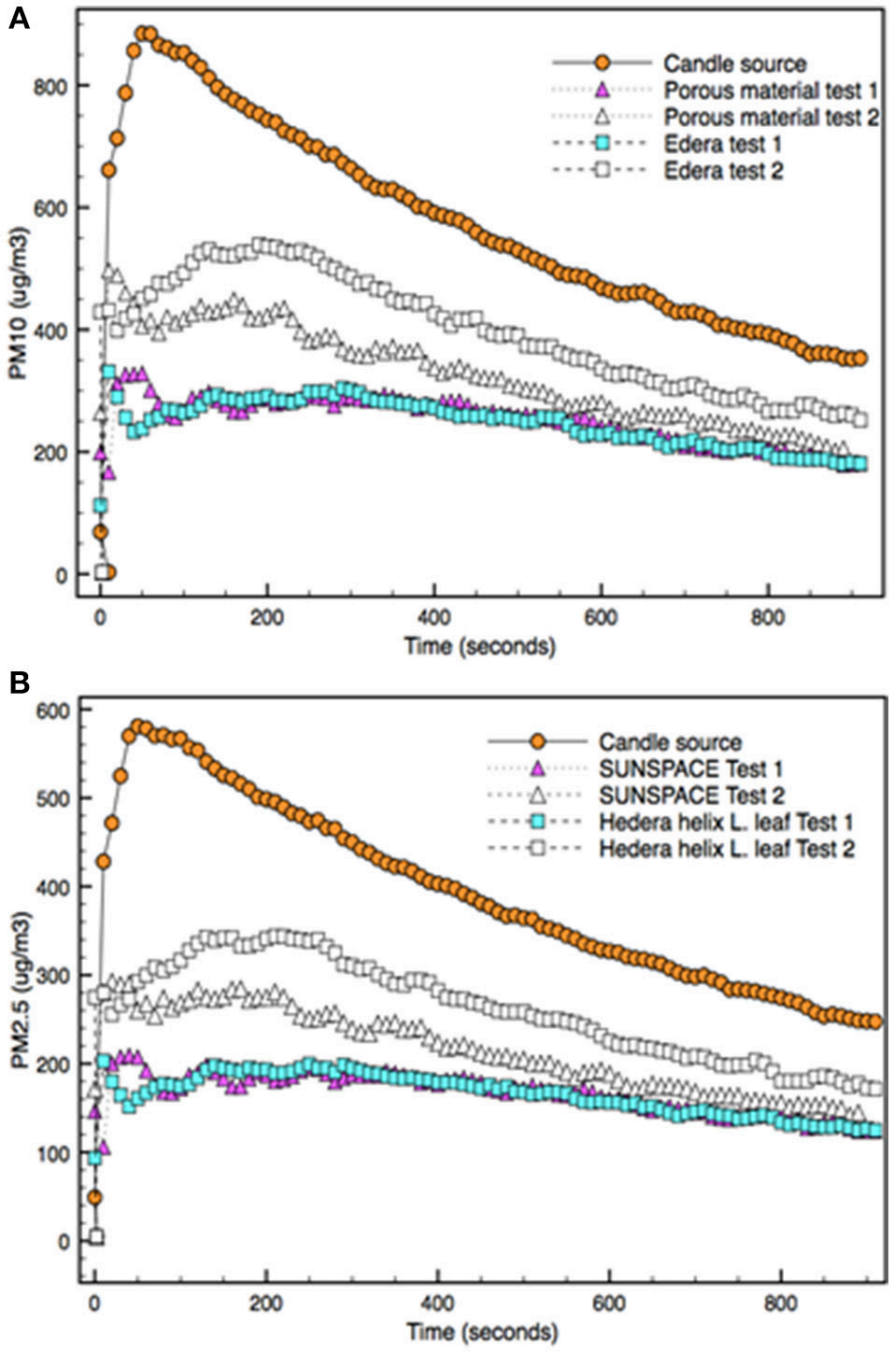
PM_10_ **(A)** and PM_2.5_ **(B)** concentrations emitted by the candle burning source in the experimental box and detected when a trapping material (*Hedera Helix L*. leaf or SUNSPACE) is used (test 1). The trapping test was repeated for both trapping materials (test 2).

Figure 4 reports the concentration of PM_10_ (A) and PM_2.5_ (B) that are emitted by the particle source and the accumulation capability of SUNSPACE and the *Hedera Helix L*. The amount of PM generated by the emission source is shown in Figure 4. The candle emits both PM_10_ and PM_2.5_, with concentrations of about 900 μg/m^3^ and at least 580 μg/m^3^ respectively.

It is also possible to follow the change in PM concentration, during the time. When the emitting source is extinguished, air PM deposits in the box, by dry deposition mechanism, mainly depending on particles mass. The two curves (PM_2.5_ and PM_10_) behavior seems quite similar. The comparison of emitted particles concentration shows that about 65% of particles consist in PM_2.5_. From the same dataset the dry velocity of the PM reduction is calculated about 0.59 μg/(s·m^3^) and 0.36 μg/(s·m^3^) for PM_10_ and PM_2.5_, respectively. As expected, particles with high sizes deposited at a higher velocity in comparison to particles with lower dimensions. As reported in literature, coarse particles (with diameters > 2.5 μm) are generally removed in a few hours from the atmosphere through dry and wet deposition. On the contrary, fine (and ultrafine) particles may persist for longer times (Brauer et al., 1989).

During Test 1 a trapping material (SUNSPACE and *Hedera Helix L*. leaf) was considered, as described in the experimental section. During Test 2, the same experiment was repeated with the same material used in Test 1 (for both materials).

Figure 4 shows that during Tests 1 and 2 the concentration of the PM detected in the box is always lower with respect to the amount of PM_10_ and PM_2.5_ emitted by the candle alone (in the preliminary experiment). Then, Test 1 allows to conclude that both SUNSPACE and *Hedera Helix L*. leaf have the capacity of trapping PM. In addition, during Test 1, the concentration of PM_10_ and PM_2.5_ detected in the box are almost the same considering the two different trapping materials, during all the acquisition time. It is possible to conclude that, in respect to the emitted PM, the two trapping materials appear to capture about the same amount of PM. Indeed, PM_10_ and PM_2.5_ concentration decreases about 3.4 and 2.9 times, respectively for both materials, in comparison to the preliminary test (where no trapping material was used). This test shows that PM particles can be accumulated by SUNSPACE, just as they are trapped by the leaf, that is currently considered the most effective method to reduce PM in urban area.

This can be attributed to new material surface morphology, with pores able to trap fine particles.

On the contrary Test 2 shows different capability in PM accumulation by SUNSPACE and *Hedera Helix L*. leaf. Figure 4 highlights that the concentration of PM_10_ and PM_2.5_ detected in the box are different, with regard to experiments made in Test 1. In particular, the concentration of PM is always higher in presence of *Hedera Helix L*. than SUNSPACE.

Then, data collected on Test 2 show that despite of the reduction in entrapment capability of both materials with time, possibly because of pore (partial) saturation, SUNSPACE seems to be more efficient compared to *Hedera Helix L*. Indeed, literature reports that the foliar PM accumulation does not necessarily increase with time (Chen et al., 2017), because the maximum loading capacity of leaves can be reached and the leaf surface may rapidly saturate (Liu et al., 2013). It is also reported that leaves PM accumulation is a dynamic process: leaves may capture PM for some time, then an event (as for example wind) can promote the release back into the air (Chen et al., 2017).

On the contrary, mainly due to the ink-bottle shaped pores (Zanoletti et al., 2018b), it was shown that new porous material can accumulate till to about 24 (±6) g/m^2^ of air PM, with dimensions lower than 2.5 μm (Zanoletti et al., 2018a).

It is evident that the highly rough and porous surface of SUNSPACE is suitable for PM trapping. Indeed, similar considerations are reported in literature for foliage: an increased roughness due to leaf hairs, scales, glands, furrows, and veins, is known to increase the PM accumulation (Blanusa et al., 2015). The best performance of SUNSPACE can be attributed to the ink-bottle pores shape and their distribution through the material depth. This represents a fundamental difference between leaves and SUNSPACE, where not only the surface is involved in the PM capture, but also the bulk, due to the pores able to entrap small particles. It was recently shown (Zanoletti et al., 2018a) that SUNSPACE sample can be washed (for example by distilled water), to remove the trapped particles. This allowed to suppose that material regeneration can be easily obtained by rainfall washing, as it occurs in nature for leaves. However work is in progress to better understand this point.

To test the capability in ultrafine particles trapping, and to verify the particles penetration depth into SUNSPACE, a sample is put in contact with titania nanoparticles (see the experimental section). The idea was not only to verify the already demonstrated (Zanoletti et al., 2018a) trapping capability but also to better understand the possibility to entrap particles with diameters in the range of few nanometers and the thickness of the material involved in their capture. In particular, the use of an incense burning source produced the porous material coating by ultrafine emitted particles, with no possibility to investigate the nanoparticle penetration into the pores (Zanoletti et al., 2018a).

In this work, the use of titanium dioxide nanoparticles would allow to discriminate the porous material and nanoparticles in SEM and TEM analysis.

A SUNSPACE sample, previously put into contact with titania nanoparticles, was analyzed by TEM and SEM.

**Figure 5:**
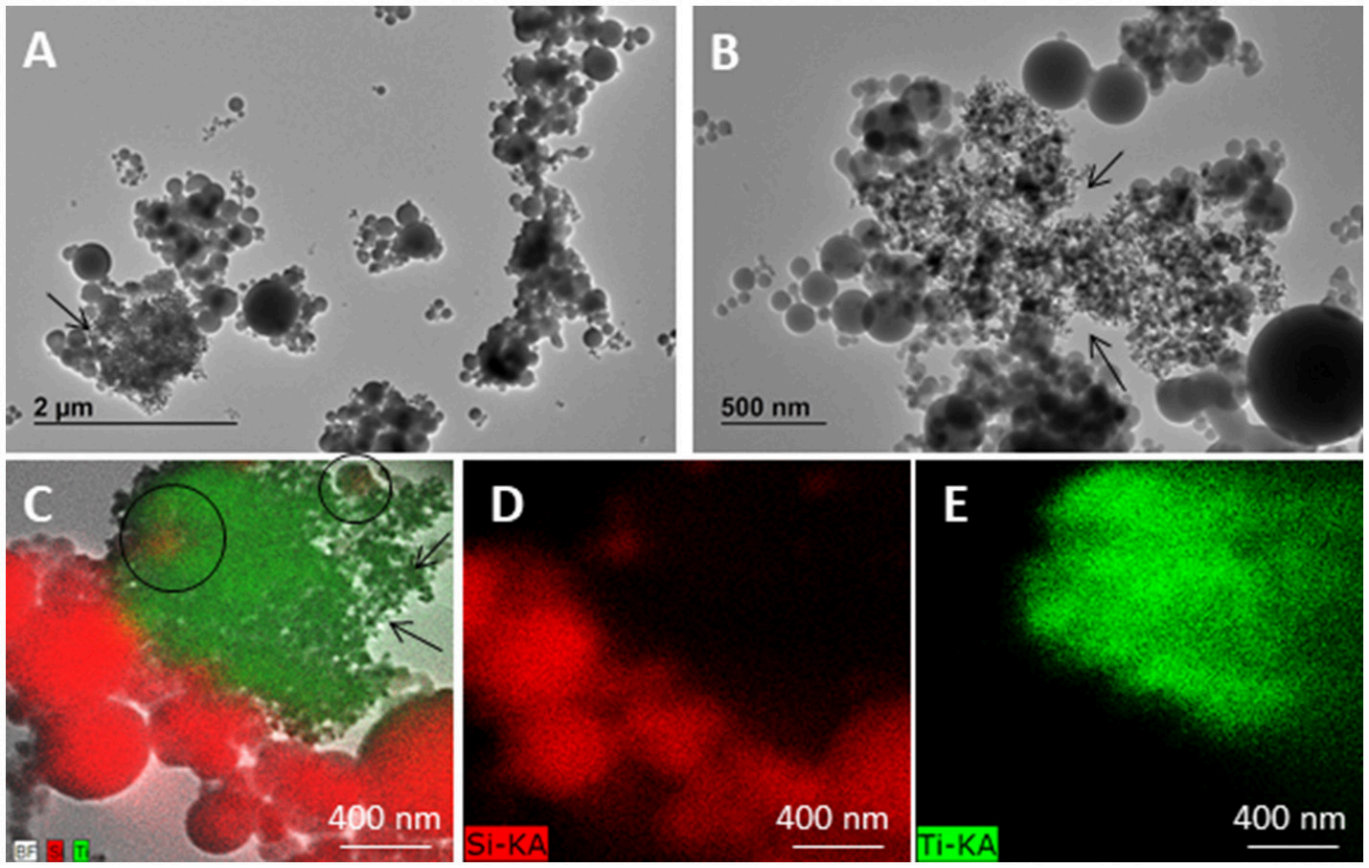
TEM (120 kV) images of SUNSPACE sample exposed to JRCNM1005a titanium dioxide nanoparticles (arrows) **(A,B)**. Elemental analysis in STEM mode at 120 kV **(C–E)**. Titanium dioxide nanoparticles (green) interact and are co-localized (circle) with SUNSPACE material (silicium, red) only.

TEM images reported in Figure 5 clearly allow to distinguish titania nanoparticles (see the arrows), having 25 nm mean size diameter, significantly lower than that of SUNSPACE particles. A comparison between TEM images reported in Figures 2, 5 further emphasizes the different contributions. It is fundamental to remember that TEM sample preparation needs sample destruction to increase the probability to find nano-materials, then also pores can be broken during the preparation. However, TEM images show that titania nanoparticles can be always found in the proximity of silica and often co-localized (Figure 5C). In particular, the image reported in Figure 5B seems to show that titania fill the SUNSPACE voids, strongly suggesting the capability of the material in trapping. Chemical analysis was also made by EDX maps of Si and Ti, reported in Figures 5C–E clearly allow to discriminate SUNSPACE and the titania nanoparticles.

**Figure 6:**
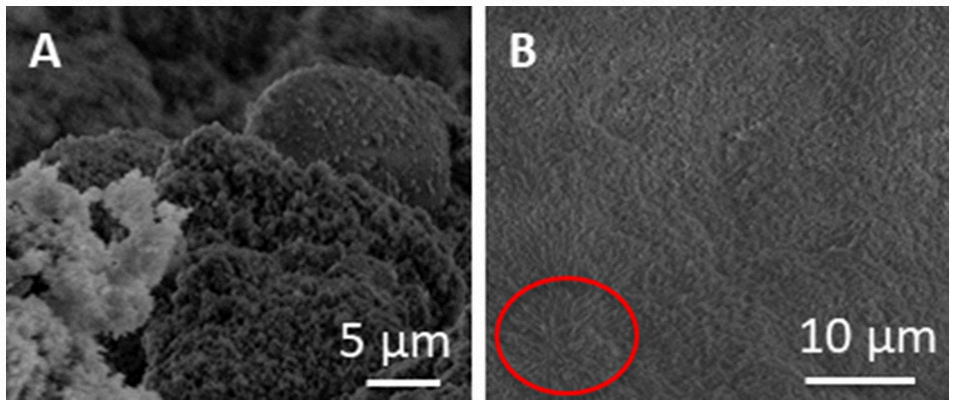
**(A,B)** SEM images of SUNSPACE sample after titania nanoparticles capture, at different magnification. The circle **(B)** highlights the iodine salts presence.

Figure 6 shows the SEM images of the same samples analyzed by TEM (Figure 5) in cross-section at different magnifications. The sponge-like structure of the material is even more evident with the presence of some needle-like structures (highlighted by a red circle), that were attributed to iodine salts (Zanoletti et al., 2018b), formed during SUNSPACE synthesis, that can be removed by rinsed sample after the synthesis.

Rinsing the sample, indeed, proved to remove soluble iodine salts, that crystallize in the structure voids. In particular, it was shown that salts removal increases the SUNSPACE porosity, by dissolution of the needle-like structures (Zanoletti et al., 2018b) filling the materials pores. In the present case, the rinsed procedure was not completely effective for the removal of all the soluble salts.

Cross-sectional sample observation is fundamental to verify the titania nanoparticles infiltration in the pores. Indeed, due to the very low dimensions of nanoparticles, morphological evidence of titania presence is extremely hard to highlight by SEM and TEM. As a consequence, it is also fundamental to use chemical analysis to search for Ti presence. Different elements were detected, such as Si, C, Na, I, and Ti.

**Figure 7:**
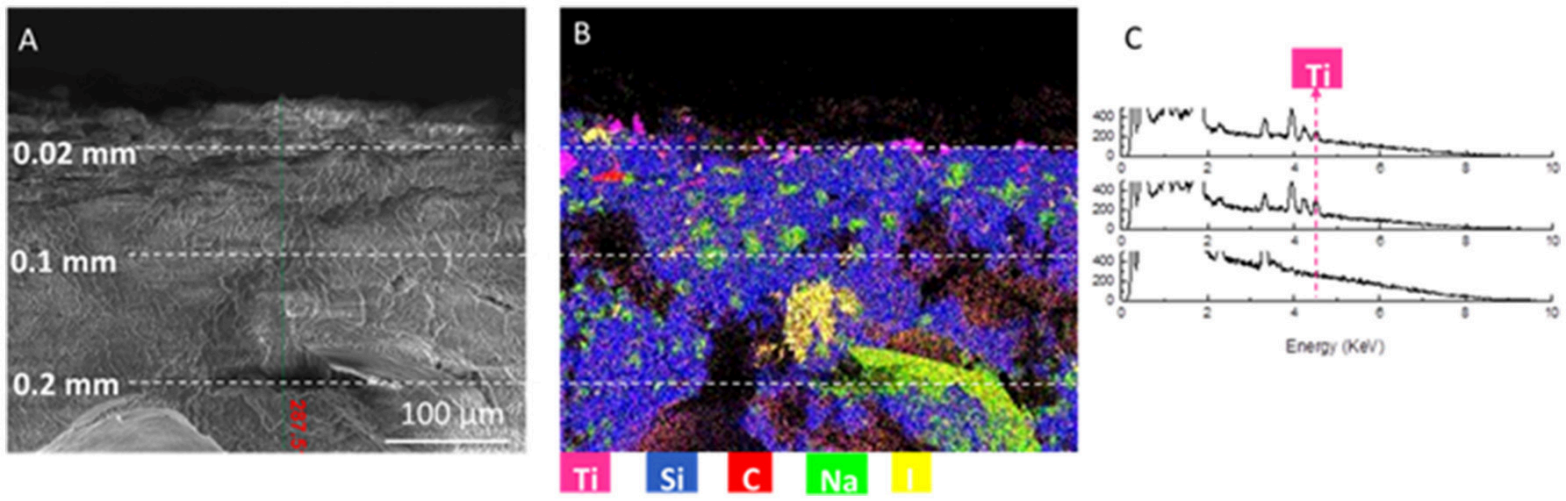
**(A)** SEM image of the cross-section of the sample, at a depth of 0.28 mm **(B)** corresponding elemental composition map with color code. Titanium signal is represented by magenta **(C)** corresponding EDS spectra at 0.02 mm from surface, 0.04 mm from surface and 0.1 mm from surface.

Figure 7 shows a SEM image taken in cross-section (Figure 7A), and corresponding EDS analysis elements map (Figure 7B).

Sodium and iodine (see Figure 7B) can be associated to synthesis residuals. Indeed, it was shown (Zanoletti et al., 2018b) that a sodium iodate hydrate phase was obtained by the dissolution of sodium ions from alginate and the presence in the solution of iodate ions from calcium iodate used as a cross-linker. Moreover, sodium may be attributed to the residue of sodium bicarbonate, used as pores forming agent. Ti can be also found in the sample cross section, due to the nanoparticles penetration along the material pores. Figure 7C shows EDS spectra collected at 0.02, 0.04, and 0.1 mm from the SUNSPACE surface. In particular, the Ti peak is highlighted. It results that titanium dioxide accumulate on the sample surface and on pores located at about 0.04 mm from the sample surface.

**Figure 8:**
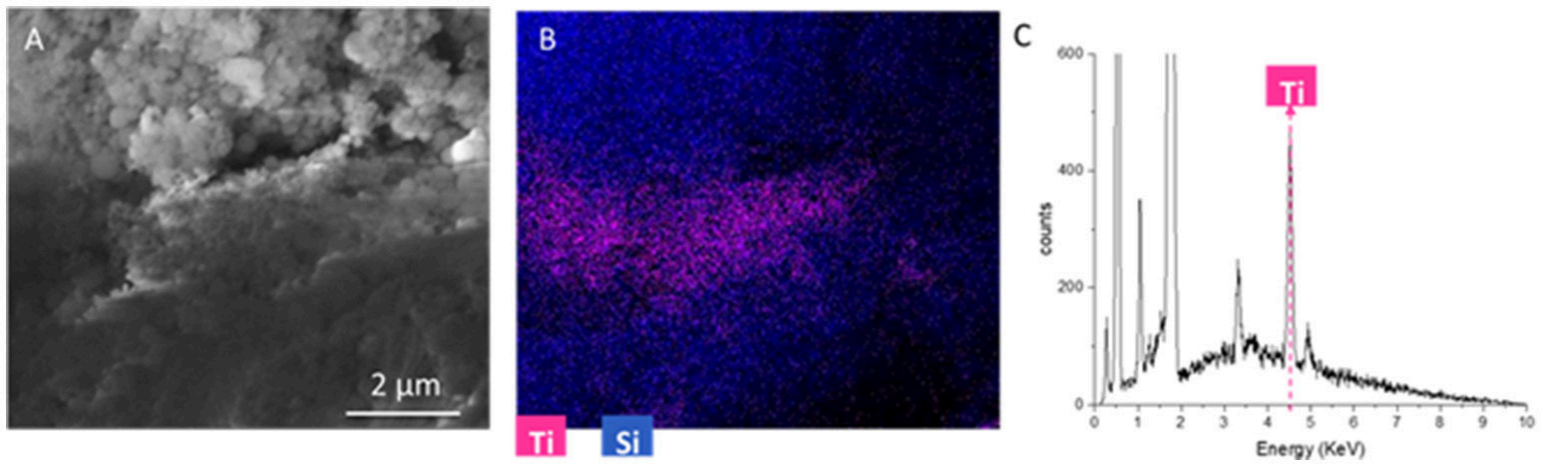
**(A)** 30000X SEM image of the cross-section of the SUNSPACE sample, at a depth of 0.045 mm from the surface **(B)** corresponding elemental composition map with color code. Titanium signal is represented by magenta **(C)** EDS spectrum of the volume with high Titanium content.

The analysis of single elements distribution (not reported here) shows that Ti and I are often correlated. This is impressive, because this correlation demonstrates that titania nanoparticles are located in pores were originally iodine salts were present. Indeed, iodine presence could be associated to the sodium iodate hydrate phase (that is generally dissolved by rinsing the sample, to remove synthesis residuals). As a consequence, it is possible to suppose SUNSPACE pores are originated not only by sodium bicarbonate decomposition, but also by SUNSPACE rinsing, because of the removal of soluble salts. Then, it is possible to conclude that after the washing procedure the pores are available for particles trapping. To better highlight the Ti spatial localization along the SUNSPACE, Figure 8 reports a SEM image collected at 30000X, in cross section, with corresponding spatial distribution of Si and Ti (Figure 8B). Ti appears to be concentrated at a depth of about 0.045 mm from the surface, as also confirmed by the EDS spectrum (Figure 8C), collected on the volume with high Ti signal, showing the high Ti contribution. This allows to conclude that the high capability in fine and ultrafine PM trapping of the SUNSPACE can be also attributed to pores present along the sample depth.

**Figure 9:**
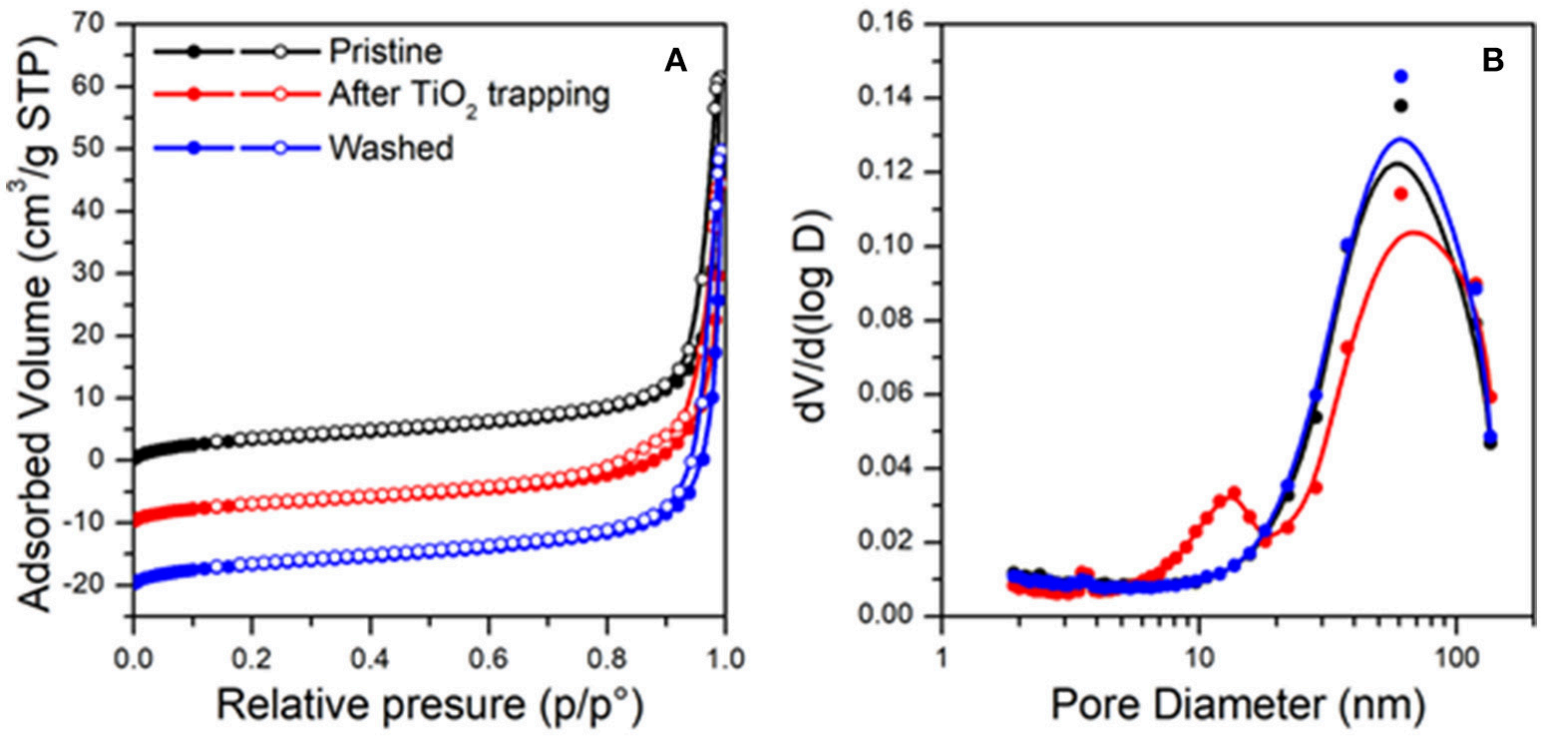
Result from N_2_ physisorption at the liquid temperature: physisorption isotherms **(A)** and BJH pore size distributions calculated from the desorption branches **(B)**.

The textural properties of the analyzed material (pristine, after TiO_2_ trapping and washed) were analyzed by N_2_ physisorption at the liquid nitrogen temperature (see Figure 9). All the samples present a Type IV isotherms, typical of mesoporous materials (Figure 9A) (Sing et al., 1985), exhibiting specific surface areas around 12 m^2^/g. For all the samples, pore size distributions calculated following the method of Barrett, Joyner, and Halenda (BJH method) (Sing et al., 1985) from the desorption branch of the isotherms show relative maxima at lower values with respect to those calculated from the correspondent adsorption branch. This is in agreement with formation of ink-bottle shaped pores (Zanoletti et al., 2018b), as revealed also by SEM analysis (Figures 7, 8). The pore size distributions calculated from the adsorption branches show maxima above 200 nm for all the samples, indicating that the internal part of pores is not significantly modified by the presence of TiO_2_ nanoparticles. On the contrary significant differences were found considering the pores distribution calculated from desorption branches (Figure 9B). In particular, the pristine material presents a maximum around 65 nm and a total pore volume of 0.096 cm^3^/g. After TiO_2_ trapping, a new relative maximum appears, resulting in a bimodal distribution of pores. A reduction of pore volume is observed (0.083 cm^3^/g). These results can be related to the partial occlusion of the pores apertures resulting from the nanoparticles accumulation. Notably, such differences cannot be highlighted by SEM and TEM analysis. Finally, after washing of the material, the textural properties of the pristine materials are recovered (one single maximum around 65 nm and a total pore volume of 0.108 cm^3^/g).

Data on PM abatement of SUNSPACE under real environmental conditions are under evaluation.

## Conclusions

This paper shows the efficacy in coarse and fine PM trapping of a new porous material, called SUNSPACE, synthesized with the aim to reduce dispersed PM in urban areas. The capability in PM_2.5_ and PM_10_ capture emitted from a candle burning is shown, also in comparison to a *Hedera Helix L*. leaf.

To investigate the possibility to sequestrate also ultrafine PM, and to investigate the nanoparticles penetration depth into the SUNSPACE, titania particles with a mean size diameter of 25 nm were used.

The SUNSPACE morphology, investigated by means of TEM and SEM analyses, revealed a porous structure. Chemical analysis of the material demonstrated its capability to entrap nanoparticles till to about 0.04 mm in sample depth. Washing test showed that the material can be also regenerated.

The potentialities of the new material to reduce air pollution, by its use as a coating on all cities surfaces (for example on walls, roofs and so on) appear very promising.

On the basis of the present results it can be suggested to use SUNSPACE to reduce PM dispersed in the air.

## Author contributions

EB, RS, JP, and LD conceived and designed the experiments. AZ and EB performed the material synthesis. AZ, FB, and AF performed the experiments. EB and LB contributed reagents, materials, analysis tools. AV and JP provided SEM and TEM characterization. TM performed Nitrogen (N_2_) physisorption experiments. EB, AZ, FB, JP, and AV wrote the paper. All authors discussed the results and contributed to the final manuscript.

### Conflict of interest statement

The authors declare that the research was conducted in the absence of any commercial or financial relationships that could be construed as a potential conflict of interest.
